# Differential requirement for Hoxa9 in the development and differentiation of B, NK, and DC-lineage cells from Flt3+ multipotential progenitors

**DOI:** 10.1186/1471-2172-14-5

**Published:** 2013-01-30

**Authors:** Kimberly Gwin, Joseph J Dolence, Mariya B Shapiro, Kay L Medina

**Affiliations:** 1 Department of Immunology, College of Medicine, Mayo Clinic, 200 First Street SW, Rochester, MN, 55905, USA

**Keywords:** Hoxa9, Flt3, IL-7R, B-cell, NK-cell, DC, Development

## Abstract

Hoxa9 is a homeodomain transcription factor important for the generation of Flt3+hiIL-7R- lymphoid biased-multipotential progenitors, Flt3+IL-7R+ common lymphoid progenitors (CLPs), and B cell precursors (BCP) in bone marrow (BM). In addition to B-cell, Flt3+IL-7R+ CLPs possess NK and DC developmental potentials, although DCs arise from Flt3+IL-7R- myeloid progenitors as well. In this study, we investigated the requirement for Hoxa9, from Flt3+ or Flt3- progenitor subsets, in the development of NK and DC lineage cells in BM. Flt3+IL-7R+Ly6D- CLPs and their Flt3+IL-7R+Ly6D+ B lineage-restricted progeny (BLP) were significantly reduced in *hoxa9−/−* mice. Interestingly, the reduction in Flt3+IL-7R+ CLPs in *hoxa9−/−* mice had no impact on the generation of NK precursor (NKP) subsets, the differentiation of NKP into mature NK cells, or NK homeostasis. Similarly, percentages and numbers of common dendritic progenitors (CDP), as well as their plasmacytoid or conventional dendritic cell progeny in *hoxa9−/−* mice were comparable to wildtype. These findings reveal distinct requirements for Hoxa9 or Hoxa9/Flt3 molecular circuits in regulation of B versus NK and DC development in BM.

## Background

Cell fate decisions in the hematopoietic system are contingent upon the concerted activities of transcription factors that activate and/or repress lineage specific developmental programs and signaling molecules that regulate the temporal expression and activation status of lineage instructing regulatory proteins. Hoxa9 is a homeobox transcription factor with key roles in embryogenesis, hematopoiesis, and leukemogenesis [1]. Hoxa9 is highly expressed in primitive blood cells in mouse and man, and downregulated as multipotent hematopoietic progenitors undergo lineage restriction and commitment [2-4]. The nonredundant role of Hoxa9 in hematopoietic progenitor biology has been determined through a variety of loss- and gain-of-function strategies. *Hoxa9−/−* mice are viable, but exhibit multiple hematopoietic defects including impaired recovery from hematopoietic stress, poor competitive reconstitution upon transplantation, impaired responses to lineage determining cytokines, and significant reductions in select LSK + subsets, myeloid precursor subsets, common lymphoid progenitors (CLP), thymic progenitors, and B cell precursors (BCP) [5-7]. Hoxa9 regulates the hematopoietic progenitor and BCP pool, in part, through transcriptional activation of the gene encoding Flt3 [4]. The Flt3 signaling pathway is critical for regulation of the hematopoietic progenitor pool and is essential at various stages for B, DC, and NK development and/or homeostasis [4,8,9].

Downregulation of c-kit, the receptor for the hematopoietic cytokine stem cell factor, accompanies lymphoid and DC lineage restriction from Flt3+hi LMPP [10-12]. Lin-c-kit+lo Flt3+ IL-7R+CLPs are a heterogeneous population that exhibit B, NK, T, and DC lineage differentiation potentials. Gene-targeted deletion of *hoxa9* significantly reduces numbers of Flt3+ IL-7R+CLPs and BCP in BM [8,13-15]. NKP are also the progeny of Flt3+ IL-7R+CLPs. Importantly, two independent groups using different experimental approaches recently showed that hematopoietic progenitors enriched for NK potential were IL-7R+, but Flt3-. In both studies, downregulation of Flt3 and upregulation of CD122 (IL-15Rα) were sequential steps in NK commitment from Flt3+ IL-7R+CLPs [16,17]. CD122 is a critical component of the IL-15R complex and IL-15 signaling is essential for NK development [18]. Lin-CD122+ NKP expressed high levels of Id2, a transcription factor important for NK differentiation [16]. Flt3 signaling has been suggested to complement IL-15 in regulation of the BM NK cell pool as well as NK homeostasis in the spleen [19]. At present, there is no information regarding the consequence of Hoxa9-deficiency, through Flt3-dependent or Flt3-independent regulatory circuits, on NK commitment, differentiation, or homeostasis.

Dendritic cells (DC) are essential innate immune effector cells. DCs are generated in BM from both the myeloid and lymphoid progenitor pathways. Common dendritic progenitors (CDPs) originate from Flt3+ myeloid progenitors that express Flt3+ but lack expression of the IL-7R [12]. IL-7R+Flt3+ CLP primarily give rise to plasmacytoid DC [20]. Flt3 signaling is essential in regulation of DC development in BM as well as DC homeostasis in the peripheral lymphoid organs [9,12,21]. The Ets-family transcription factor PU.1 regulates Flt3 in DCs [22]. Interestingly, a recent study characterizing Hoxa9 target genes found many Hoxa9 binding sites were also bound by PU.1, notably *flt3*[23]. Therefore, a molecular contribution of Hoxa9 to the development and/or homeostasis of DC lineage cells, merits further investigation.

The goal of this study was to examine the role of Hoxa9 in regulation of NK and DC development and differentiation. We found that, in contrast to B cells, Hoxa9 is largely dispensable for the development or peripheral homeostasis of NK or DC lineage cells. These findings uncover a distinct requirement for Hoxa9 in regulation of B versus NK and DC development from Flt3+ MPPs.

## Methods

### Mice

C57Bl6 mice (8–16 weeks of age) were purchased from The Jackson Laboratory (Bar Harbor, ME). *Hoxa9−/−* and *flt3l−/−* mice have been described [4] and were bred and maintained at the Mayo Clinic animal facility and used between 8–16 weeks of age. All mice were aged matched in individual experiments and the experimental data did not vary as a function of age or sex. Bone marrow cells from individual male or female mice were collected from the four hind limb bones after carbon dioxide asphyxiation and single cell suspensions made in preparation for flow cytometric analysis. All experiments were carried out in accordance with Mayo Clinic Institutional Animal Care and Use Committee guidelines under Protocol A17509.

### Antibodies and flow cytometry

**Figure 1:**
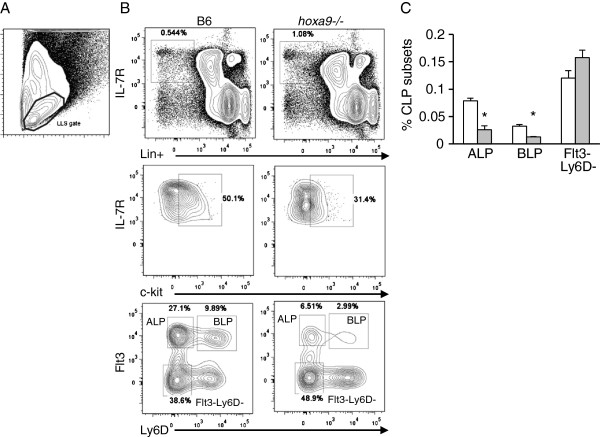
**Reduction in lymphoid progenitor subsets in *hoxa9−/−*mice. A**) Lymphoid light scatter gate (LLS) used throughout these studies. **B**) Flow cytometric analysis of BM Lin-IL-7R+c-kitlo CLP subsets in wildtype B6 and *hoxa9−/−* mice. The gating strategy is depicted from top to bottom with arrows. The lineage cocktail includes antibodies to CD45R/B220, CDllb/Mac1, Ly6G/Gr1, TER119, CD3ε, CD8α, CD11c, NK1.1, and Ly6C. Lin-IL-7R+ cells (top panel) were gated for IL-7R+c-kitlo cells (middle panel) and Lin-IL-7R+c-kitlo cells were fractionated into 3 subsets based on differential expression of Flt3 and Ly6D. ALPs are gated as Lin-IL-7R+c-kitloFlt3+Ly6D- and BLP as Lin-IL-7R+c-kitloFlt3+Ly6D+. A third Lin-IL-7R+c-kitloFlt3-Ly6D- subset is labeled as Flt3-Ly6D-. **C**) Summary of ALP, BLP, and Flt3-Ly6D- CLPs precursor frequencies in B6 (white bars) and *hoxa9−/−* (gray bars) mice. Precursor frequencies were calculated by multiplying sequential percentages of each gated region (ie., % Lin-IL-7R + in LLS gate x % ckitlo x %’s of ALP, BLP, or Flt3-Ly6D-). Data is representative of 5 individual B6 and *hoxa9−/−* animal BM analyses. The asterisk indicates p < 0.05. Error bars represent SEM.

Methods for flow cytometry and progenitor isolation have been extensively described [4,13]. Flow cytometric analysis was performed on the FACS-Canto or LSRII cytometers (BD Biosciences, San Jose, CA) and analyzed using FlowJo software (Tree Star, Ashland, OR). All antibodies used in this study were purchased from eBioscience, BioLegend, or Pharmingen. Antibody conjugations and combinations used to delineate progenitor subsets were the following: ALP/BLP stain Lin+ cocktail (FITC conjugated CD45R/B220, CDllb/Mac1, Ly6G/Gr1, TER119, CD3ε, CD8α, CD11c, NK1.1, Ly6C), c-kit APCefluor 780, Sca1-PerCP Cy5.5, biotin IL-7R (visualized with streptavidin PeCy7), Flt3 PE, and Ly6D APC; NKP stain Lin+ cocktail (APC conjugated CDllb/Mac1, CD3ε, NK1.1, CD19, Ly6D), biotin CD244 (visualized with streptavidin PeCy7), CD27 APCefluor 780, IL-7R PE, Flt3 PerCPefluor 710, and CD122 FITC; and CDP stain Lin+ cocktail (FITC conjugated CD45R/B220, CDllb/Mac1, Ly6G/Gr1, TER-119, CD3ε, CD8α, NK1.1), c-kit APCefluor 780, Flt3-PE, bio-IL-7R (visualized with streptavidin PeCy7), and CD115-APC; NK subsets, CD3ε PeCy7, CD122 FITC, NK1.1 AF780, and Dx5 APC; DC subsets, B220 AF780, MHCII APC, and CD11c PerCPCy5.5. All flow cytometric analysis depicts progenitor subsets within the lymphocyte light scatter gate (LLS) of BM (Figure 1A).

### Statistics

Statistical significance was determined using the Student-t test and p-values less than 0.05 were considered significant.

## Results

### Selective reduction in Flt3+IL-7R+, but not Flt3-IL-7R+CLPs in *hoxa9−/−* mice

We previously showed that Lin-c-kitloIL-7R+Flt3+ CLPs are significantly reduced in *hoxa9−/−* mice [4]. Lin-c-kitloFlt3+ IL-7R+cells can be fractionated into Flt3+IL-7R+Ly6D- all lymphoid progenitors (ALP) and Flt3+IL-7R+Ly6D+B lineage restricted progenitors (BLP) [24]. Therefore, we employed this flow cytometric analysis to further define the lymphoid/B lineage developmental block in *hoxa9−/−* mice [4,15]. The Lin+ cocktail used was extended to include antibodies to CD11c, NK1.1, and Ly6C to eliminate contamination of CLPs with mature NK1.1+ NK or CD11c/Ly6C + DCs. First, Lin-IL-7R+ cells within the lymphocyte light scatter gate (Figure 1A) were gated for low expression of c-kit (Figure 1B, gated region, middle panel). We note that using the extended Lin+ cocktail and gating on IL-7R+ cells, that overall percentages of Lin-IL-7R+ were not reduced in *hoxa9−/−* mice (0.71 ± 0.20% vs. 0.83 ± 0.29% in *hoxa9−/−* vs. wildtype, respectively, n = 5 mice). The Lin-IL-7R+c-kitlo subset was then fractionated into ALP and BLP using differential expression of Flt3 and Ly6D (Figure 1B, bottom panels). This gating strategy revealed that ALP, as well as their B lineage restricted progeny BLP, are significantly reduced in *hoxa9−/−* mice, consistent with our previous findings [4]. Precursor frequency analysis revealed that ALP represent 0.061 ± 0.013% vs. 0.024 ± 0.01% of BM cells within the lymphoid light scatter gate in B6 vs. *hoxa9−/−* mice, respectively (p = 0.0009). Similarly, BLP represent 0.026 ± 0.002% vs. 0.01 ± 0.002% of BM cells within the lymphoid light scatter gate in B6 vs. *hoxa9−/−* mice, respectively (p = 0.0005). Less BLP resulted in reductions in CD19+IgM- B cell precursors (11.5 ± 2% vs. 20.03 ± 2.0% of BM cells within the lymphoid light scatter gate, p = 0.0009, in *hoxa9−/−* vs. B6, respectively), consistent with our previous report [4,15]. In contrast, a conspicuous population of IL-7R+Flt3-Ly6D- CLPs was not reduced (0.087 ± 0.22% vs. 0.11 ± 0.06% of BM cells within the lymphoid light scatter gate in B6 vs. *hoxa9−/−*mice, respectively, p = 0.11). Percentages of ALP, BLP, and IL-7R+Flt3-Ly6D- CLPs in B6 control and *hoxa9−/−* mice are summarized in Figure 1C (data represents mean and SEM). We conclude from these flow cytometry analyses that Hoxa9-deficiency preferentially reduces Flt3+IL-7R+, but not Flt3-IL-7R+ CLP subsets.

### NK development and differentiation in *hoxa9−/−* mice

CD27+ and CD244+ are cell surface markers expressed on IL-7R-Flt3+ multipotential progenitors (MPP) and CLPs, and maintained on mature NK cells [25,26]. A small subset of CD27+CD244+IL-7R+ progenitors were identified in BM that lacked expression of surface markers displayed by lineage committed precursor subsets, including CD19, Mac1, Ly6D, NK1.1, and CD3. CD122 (IL-2Rβ) is a critical component of the IL-15R complex (comprised of IL-15Rα, IL-2Rγ and IL-2Rβ) [27]. Expression of the IL-15R is acquired as CLP differentiate into NK cells and is associated with loss of B, T, and DC lineage differentiation potentials. Fathman, et al., evaluated NK precursor potential in Lin- (CD19- Mac1- Ly6D- NK1.1- CD3-) CD27+CD244+IL-7R+ cells [17]. Within the Lin-CD27+CD244+IL-7R+ subset, differential expression of Flt3 and CD122 distinguished three progenitor subsets, Flt3+CD122-, Flt3-CD122-, and Flt3-CD122+. Transplantation studies revealed that the Lin- CD27+CD244+ IL-7R+Flt3+ subset were found to functionally largely overlap CLPs, while the Lin-CD27+ and Lin-CD244+ subsets gave rise to NK cells. *In vitro* differentiation studies confirmed that the Lin-CD27+CD244+IL-7R+Flt3-CD122- and CD122+ subsets generated only NK cells. Based on differential expression of CD122 and the NK lineage bias exhibited by these cells in functional assays, the Lin-CD27+CD244+IL-7R+Flt3-CD122- subset was designated PreNKP and the Lin-CD27+CD244+IL-7R+Flt3-CD122+ subset rNKP [17]. Previous studies showed that loss of Flt3-L significantly reduced the mature BM NK pool [9,19]. However, a role for Hoxa9, through Flt3 dependent or independent mechanisms, in regulation of NK commitment and differentiation from the CLP stage has not been determined.

**Figure 2:**
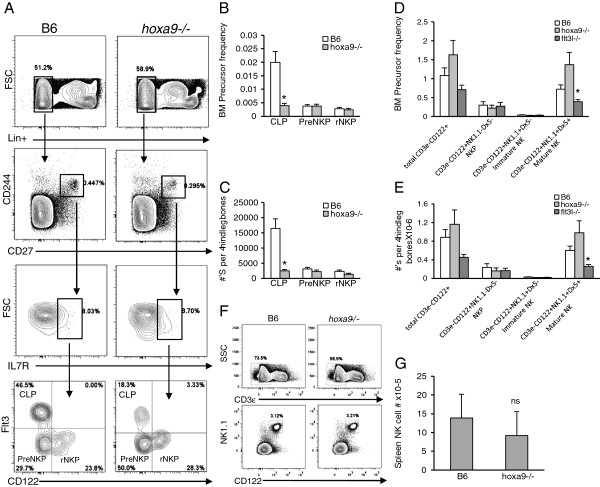
**Hoxa9 is dispensable for NK differentiation and homeostasis. A**) BM cells from wildtype B6 or *hoxa9−/−* mice were isolated and stained with combinations of antibodies as detailed in the Methods to resolve PreNKP and rNKP progenitor subsets. The Lin+ antibody cocktail includes CDllb/Mac1, CD3ε, NK1.1, CD19, and Ly6D. The gating strategy is depicted from top to bottom with arrows. Data is representative of 6 individual mice of each genotype. **B**) NKP subset frequencies in BM of wildtype B6 (open bars) and *hoxa9−/−* (gray bars) mice. Precursor frequencies reflect percentages of CLP, PreNKP, or rNKP within the Lin-CD244+CD27+IL-7R+ gated fractions. **C**) Absolute numbers of BM NKP subsets in wildtype B6 (open bars) and *hoxa9−/−* (gray bars) mice. **D**) Summary of frequencies or **E**) absolute numbers of BM total CD3ε-CD122+ progenitors, NKP (CD3ε- CD122+NK1.1-Dx5-), immature NK (CD3ε-CD122+NK1.1+ Dx5-), and mature NK (CD3ε-CD122+NK1.1+ Dx5+) cells in wildtype B6 (open bars), *hoxa9−/−* (light gray bars), and *flt3l−/−* (dark gray bars). Data reflects the average of each indicated subset pooled from analysis of 7–8 mice per genotype. **F**) Flow cytometry profile of CD3ε- NK1.1+CD122+ NK cells in spleen. Spleen mononuclear cells were first gated on CD3ε- (top panels). The CD3ε- splenocytes were then analyzed for expression NK1.1 and CD122 (bottom panels). **G**) Absolute numbers of NK cells (CD3ε-NK1.1+CD122+) in spleen. Precursor frequencies were calculated by multiplying sequential percentages of each gated region as described in Figure 1. Absolute numbers were determined by multiplying precursor frequencies x numbers of BM mononuclear cells obtained from 4 hind limb leg bones. The asterisk indicates p < 0.05. Error bars represent SEM.

We showed in Figure 1B-C that a subset of IL-7R+ CLPs that lack Flt3- and Ly6D- are spared by Hoxa9-deficiency. Since IL-7R+Flt3-Ly6D- CLPs are enriched for NK precursors we speculated that Hoxa9 might be dispensable for NK commitment and differentiation from CLPs. To make this determination, we employed the flow cytometric analysis described above to examine the NK precursor compartment in *hoxa9−/−* mice. Consistent with our findings in Figure 1B, percentages and absolute numbers of Lin- CD27+CD244+IL-7R+Flt3+ CLPs were significantly reduced in *hoxa9−/−* mice (Figure 2A-C). Importantly, shown in Figure 2A and summarized in Figures 2B-C, neither frequencies nor numbers of PreNKP or rNKP within the lymphoid light scatter gate in BM were diminished by Hoxa9-deficiency. The reduction in CLPs reinforces the requirement for Hoxa9 in regulation of the IL-7R+Flt3+ CLP subset. Surprisingly, the reduction in IL-7R+Flt3+ CLPs did not impact the generation or maintenance of Pre-NKP or rNKP. These data suggest that commitment to the NK fate is not dependent on Hoxa9 function within CLPs.

Flt3 signaling has been implicated in regulation of the mature BM NK compartment. Specifically, it was shown that mature Lin-CD122+NK1.1+Dx5+ NK cells are reduced in *flt3l−/−* mice and our results concur with those findings (Figure 2D-E) [19]. Until the identification of NKP, BM NK transitional subsets were distinguished by differential expression of CD3ε, CD122, NK1.1, and CD49b/Dx5 [28]. To determine whether Hoxa9 regulates NK differentiation from NKP, BM cells from B6 wildtype, *hoxa9−/−*, and *flt3−/−* mice were stained with antibodies to CD3ε, CD122, NK1.1, and CD49b (Dx5) [28]. As shown in Figure 2D-E, percentages and numbers of CD3ε-CD122+NK1.1-Dx5- NKP and CD3ε-CD122+NK1.1+Dx5- immature NK cells were similar between the 3 genotypes. These results suggest that Hoxa9 is dispensable for NK differentiation from rNKP. However, in contrast to Flt3 signaling, Hoxa9 is also dispensable for NK homeostasis in BM and spleen as neither mature NK cell frequencies nor numbers are reduced in BM or spleen in *hoxa9−/−* mice (Figure 2D-G).

### DC differentiation in *hoxa9−/−* mice

**Figure 3:**
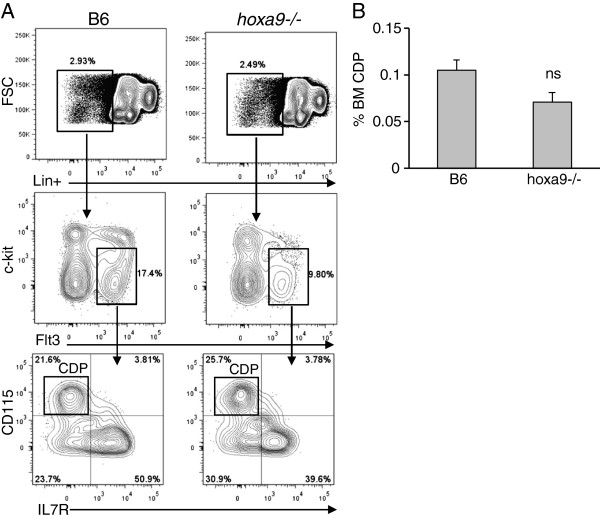
**Hoxa9 is dispensable for the development of CDP in BM. A**) Flow cytometry profile of CDP in B6 and *hoxa9−/−* mice. The Lin+ antibody cocktail included CD45R/B220, CDllb/Mac1, Ly6G/Gr1, TER-119, CD3ε, CD8α, and NK1.1. The gating strategy is depicted from top to bottom with arrows. CDP are identified as Lin-ckitloFlt3+ CD115/M-CSFR+. **B**) CDP precursor frequency. Precursor frequencies were calculated by multiplying sequential percentages of each gated region as described in Figure 1. Data is representative of 4 individual mice per genotype. Error bars represent SEM.

**Figure 4:**
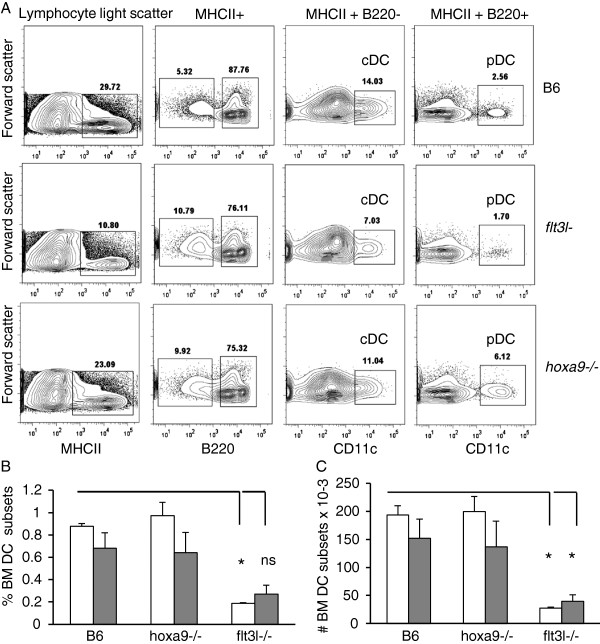
**Hoxa9 is not required for DC differentiation. A**) Flow cytometric comparison of DC subsets in BM of **wildtype** B6, *flt3l−/−*, and *hoxa9−/−* mice. BM cells were first analyzed for MHCII expression. The MHCII + cells were then fractionated based on differential expression of CD45R/B220 into MHCII+B220+ or MHCII+B220-. Conventional DCs are MHCII+B220-CD11c+. Plasmacytoid DCs are MHCII+B220+ CD11c+. **B**) pDC (white bars) and cDC (gray bars) precursor frequency in BM. **C**) Absolute numbers of pDC (white bars) and cDC (gray bars) in BM. Precursor frequencies were calculated by multiplying sequential percentages of each gated region as described in Figure 1. Absolute numbers were determined by multiplying precursor frequencies x numbers of BM mononuclear cells obtained from 4 hind limb leg bones. Data is representative of 4 mice per genotype. Error bars represent SEM.

Lineage-restricted precursors of DCs are enriched within Lin-ckit+loFlt3+ MPPs [12]. Flt3 signaling is essential for DC differentiation and homeostasis [21,29]. Lineage-restricted common dendritic progenitors CDP express the CD115/M-CSFR, but lack expression of the lymphoid-lineage-associated cytokine receptor IL-7R [12]. Lin-ckit+loFlt3+ progenitors are reduced in *hoxa9−/−* mice (Figure 3A, middle panel). However, percentages of Lin-c-kit+lo Flt3+ cells expressing the CD115/M-CSFR were comparable between wildtype and *hoxa9−/−* mice (Figure 3A and summarized in Figure 3B). Next we compared percentages and absolute numbers of DC-lineage progeny. CD11c+MHCII+B220- conventional DC (cDC) and CD11c+MHCII+B220+ plasmacytoid DC (pDC) are the progeny of CDP, although there is experimental evidence that DCs develop from CLP [20,29-31]. As shown in Figure 4A-C, frequencies and absolute numbers of cDC (gray bars) and pDC (white bars) in *hoxa9-* mice were largely comparable to WT. Furthermore, percentages and absolute numbers of cDC and pDC were also found at comparable frequencies to B6 wildtype mice in the spleen (data not shown). Consistent with previous findings, cDC and pDC were reduced in BM and spleen of *flt3l−/−* animals (Figure 4B-C and data not shown) [9]. These data suggest that Hoxa9 is not a critical component of the regulatory circuitry that orchestrates DC commitment and/or differentiation from Flt3+ MPPs.

## Discussion

In this study we investigated the role of Hoxa9 in regulation of NK- and DC-lineage commitment and differentiation in BM. Hoxa9-deficient mice exhibit reductions in select hematopoietic progenitor subsets, including Flt3+ MPPs, Flt3+IL-7R+ CLPs, myeloid progenitor subsets, and BCPs, in addition to Pro-T subsets in the thymus [4,15]. Hoxa9 regulates these hematopoietic progenitor subsets, at least in part, through transcriptional regulation of Flt3 [4]. Flt3 signaling is important for the development and/or maintenance of Flt3+ MPPs, CLPs, B, T, NK, and DC lineage cells [9,19]. Previously, a role for Hoxa9, through Flt3-dependent or independent regulatory circuits, in regulation of NK- or DC-lineage commitment and differentiation in BM had not been established. Here we show that Hoxa9 is dispensable for the generation and differentiation of NK and DC lineage cells in BM. In contrast, Hoxa9 plays a non-redundant role in regulation of Flt3+IL-7R+ CLPs, as well as their Flt3+IL-7R+Ly6D+ B-lineage restricted progeny. Importantly, the reduction in Flt3+IL-7R+ CLPs in *hoxa9−/−* mice does not compromise the generation of NKP. These findings distinguish a critical function for Hoxa9 in Flt3+IL-7R+ CLPs in regulation of B, but not NK lineage commitment.

**Figure 5:**
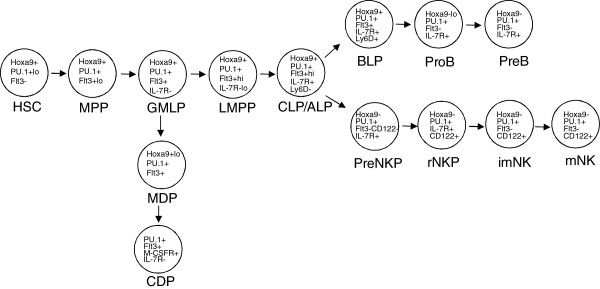
**Model depicting Hoxa9 and Flt3 expression patterns in DC, NK, and B cell precursors in BM.** HSC, MPP, GMLP and LMPP are primitive LSK+ subsets defined by differential expression of Flt3 and VCAM-1 [46]. CLP are Lin-ckitloIL-7R+Flt3+Ly6D-. BLPs are a B lineage restricted subset of CLP/ALP that express Ly6D. ProB through PreB are B220+ B cell progenitor subsets. PreNKP are Lin-CD244+CD27+IL-7R+Flt3-loCD122- and are the presumed progeny of CLP/ALP. rNKP through mNK are NK lineage restricted subsets distinguish based on expression of CD122, NK1.1 and Dx5. Hoxa9 and Flt3 expression patterns within the subsets are derived from our previous study [4], or deduced from the Immunological Genomic Project database (http://www.immgen.org/databrowser/index.html).

The differentiation of DC lineage cells from multipotent hematopoietic progenitors is driven by the combinatorial activities of cytokines, notably Flt3L, M-CSF, and GM-CSF [32]. Importantly, DCs develop from both myeloid and lymphoid precursors. Myeloid progenitors are minimally affected by Hoxa9-deficiency, thus providing an alternate route for DC production [6]. Although Hoxa9 is a critical regulator of *flt3*, Flt3 is expressed, albeit at lower levels, in *Hoxa9-*deficient hematopoietic progenitors [4]. In addition to Hoxa9, the Ets-family transcription factor PU.1 is a key regulator of *flt3*. It was previously established that PU.1 regulation of Flt3 is essential for DC development [22]. Hoxa9 transcripts are low/negative in CDP and Macrophage Dendritic Progenitors (MDP), compared to Flt3+ MPP or CLPs. In contrast, PU.1 transcripts are elevated in CDP and MDP (Figure 5) [33]. Hierarchically, MDP give rise to CDP, and CDP are the lineage restricted precursors of conventional and plasmacytoid dendritic cells [34]. Interestingly, MDP numbers are not reduced in *flt3−/−* mice, suggesting that Flt3 expression is not required for commitment to the DC fate [21]. Our data extend these results and establish that Hoxa9 function is dispensable for commitment and differentiation in the DC lineage.

A recent fate mapping study of an IL-7R reporter mouse suggested that all NK-lineage cells progress through an IL-7R + stage [35]. IL-7R + hematopoietic progenitors are generated in *flt3−/−* and *flt3l−/−* mice, albeit at reduced frequencies [8,13]. Thus, Flt3 signaling is not essential for induction of IL-7R expression, consistent with our current findings. CD122 is upregulated in Flt3- CLPs uncoupling Flt3 signaling from induction of CD122 expression. PreNKP have been proposed to be developmentally positioned downstream of Flt3+ IL-7R+ CLPs (Figure 5). We note that the few B220+ cells identified in *FL−/− x IL-7Ra−/−* mice are NK1.1+ [36]. Furthermore, *il7−/−* mice exhibit no deficiencies in NK cells in BM [19,37]. These experimental results together with our findings herein suggest that commitment to the NK fate is not dependent on the combinatorial activities of Flt3 and/or IL-7R signaling or Hoxa9 function.

We show significantly reduced percentages of Flt3+IL-7R+ CLP/ALPs and Flt3+IL-7R+ BLPs in *hoxa9−/−* mice. The combinatorial activities of Flt3 and IL-7R signaling are indispensable for B cell genesis [36]. IL-7R signaling in CLPs promotes the generation of BLP and their subsequent differentiation into BCP through Stat5-mediated induction of the B-cell fate determinant EBF [38,39]. EBF in turn suppresses Id2 transcription [40,41]. Id2 is critical for NK differentiation and transcript levels are increased in IL-7R+Flt3- rNKP [16,42-44]. The observation that frequencies and numbers of PreNKP and rNKP are essentially unchanged while BLP are reduced suggest that Hoxa9, alone or in combination with Flt3 signaling, regulates a critical molecular circuitry that dictates the B vs. NK fate decision in Flt3+IL-7R+ CLP/ALPs.

Finally, the dramatic downregulation of Flt3 in PreNKP suggests that commitment to the NK fate might initiate a series of events that silences *flt3* expression. Induction of the B lineage commitment factor Pax5 silences Flt3 transcription in committed BCP [45]. Interestingly, failure to downregulate Flt3 impairs B cell differentiation. Ectopic expression studies examining the consequences of sustained expression of Flt3 or Hoxa9 on NK differentiation will be informative with regard to the requirement for regulated expression of Flt3 or Hoxa9 in NK development.

## Conclusion

In summary, Hoxa9 is a critical regulator of early hematopoietic differentiation, in part through regulation of Flt3. We uncovered differential requirements for a Hoxa9 in the development/maintenance of IL-7R+Flt3+ CLPs and BLP. Although NKP and CDP share a common Lin-c-kitloFlt3+ progenitor with BLP, Hoxa9 is dispensable for their development and differentiation. These findings reinforce the stringent requirement for Hoxa9 in regulation of B lymphopoiesis and support future studies aimed at delineating Hoxa9-dependent genetic circuits that dictate the B cell fate decision.

## Abbreviations

BM: Bone marrow; MPP: Multipotential progenitor; LSK: Lineage negative Sca-1+ c-kit + hi; GMLP: Granulocyte-macrophage-lymphoid progenitor; LMPP: Lymphoid biased multipotential progenitor; CLP: Common lymphoid progenitor; ALP: All lymphoid progenitor; BCP: B cell precursor; NKP: Natural killer cell progenitor; rNKP: Restricted natural killer cell progenitor; CDP: Common dendritic progenitor; MDP: Macrophage dendritic progenitor; Flt3L: Flt3 ligand.

## Competing interests

The authors declare that they have no competing financial interests.

## Authors’ contributions

KLM conceived and designed the experiments, performed data analysis, made figures and wrote the manuscript. KG, JJD, MBS designed and performed experiments and analyzed data. All authors read and approved the final manuscript.
